# Supplementary material for: The Transcription Factor MiMYB8 Suppresses Peel Coloration in Postharvest ‘Guifei’ Mango in Response to High Concentration of Exogenous Ethylene by Negatively Modulating MiPAL1
Source: Int J Mol Sci. 2024 Apr 29;25(9):4841. doi: 10.3390/ijms25094841 (PMC11084497; doi:10.3390/ijms25094841)
Supplement: Supplementary file 1 [file ijms-25-04841-s001.zip › ijms-2913186-supplementary.pdf]

Table S1 Summary of Primers Used in This Study.

Assay	Genes	Vectors	Reverse Primers（5'-3'）
	MiMYB6	none	Forward:TGGCTGCAACACAAGAAGGA
			Reverse:TTCAGTCTCGAGCCTACCCA
	MiMYB5	none	Forward:AGAAACCAAGGAACCGACCC
			Reverse:TTGGCTTTGGGAGATGGACC
	MiMYB8	none	Forward:CGGCGGTGTTAATGTGGATG
			Reverse:CTTTCGCTCCACTCTCAGGG
qRT-PCR	*MiPAL1*	none	Forward:GCTGTTGGTTCTGGCTTAGC
			Reverse:AGCACCATTCCAACCACTGA
	MiPAL2	none	Forward:AGGAGGTGGAGAGTGCTAGA
			Reverse:GCCGCCAGAGAGATTTGATG
	MiActin	none	Forward:AATGGAACTGGAATGGTCAAGGG
			Reverse:TGCCAGATCTTCTCCATGTCATCCCA
	MiMYB8	pCAMBIA1302-GFP	Forward:CCATGGATGGCGTTTAATAGAAAAGAAGTGGATCGG(NcoI)
			Reverse:ACTAGTGATCCTCTGCAGCGCGACATTT(SpeI)
	MiMYB8	none	Forward:ATGGCGTTTAATAGAAAAGAAGTGGATCGG
PCR amplification			Reverse:TCAGATCCTCTGCAGCGCGACATTT
	MiPAL1 promoter	none	Forward:CATAAGAATCATGCAAGCCATACAGAG
			Reverse:TGAACAAAGGGACGGATAGCTACG
Transient overexpression	*MiMYB8*	pGreen II 62-SK	Forward:GGATCCATGGCGTTTAATAGAAAAGAAGTGGATCGG(BamHI)
			Reverse:GAATTCTCAGATCCTCTGCAGCGCGACATTT(EcoRI)
	MiPAL1 promoter	pHIS2	Forward:GAATTCCATAAGAATCATGCAAGCCATACAGAG(EcoRI)
			Reverse:GAGCTCTGAACAAAGGGACGGATAGCTACG(SacI)
Y1H			
	MiMYB8	pGADT7	Forward:GGATCCATGGCGTTTAATAGAAAAGAAGTGGATCGG(EcoRI)
			Reverse:GAATTCTCAGATCCTCTGCAGCGCGACATTT(BamHI)
	MiPAL1 promoter	pGreen II 0800-LUC	Forward:GGTACCCATAAGAATCATGCAAGCCATACAGAG(KpnI)
			Reverse:GGATCCTGAACAAAGGGACGGATAGCTACG(BamHI)
DLR			
	MiMYB8	pGreen II 62-SK	Forward:GGATCCATGGCGTTTAATAGAAAAGAAGTGGATCGG(BamHI)
			Reverse:GAATTCTCAGATCCTCTGCAGCGCGACATTT(EcoRI)

Table S2 Nucleotide Sequences of MiPAL1, MiPAL1 Promoter and MiMYB8.

Genes	Sequence
MiPAL1	ATGTTGGTGAGAATCAACACCCTGTTACAAGGATACTCAGGTATCAGGTTTGATATCCTGGAAGCCATCACCAACTT<br>CCTTAACCATAACGTCACCCCGTGTCTGCCACTCCGTGGCACAATCACTGCATCGGGTGACCTGGTTCCGTTATCAT<br>ACATTGCCGGACTCTTGACTGGAAGGCCAAACTCGAAGGCAGTTGGGCCAAATGGGCAAGTCCTGAATGCCAATG<br>AAGCCTTTCAACTTGCTGGTATCCATGGTGGATTCTTTGAGTTGCAACCTAAAGAGGGTCTTGCTTTAGTGAATGGC<br>ACGGCTGTTGGTTCTGGCTTAGCTTCAATGGTGCTTTTTGAGGCTAACATACTTGCTATTCTATCAGAAGTTTTGTCA<br>GCAATTTTTGCAGAAGTTATGAATGGAAAACCTGAGTTTACTGATCACTTGACACATAAATTGAAGCACCATCCGGG<br>CCAAATTGAAGCTGCAGCTATCATGGAGCACATTTTAGCTGGCAGCAGTTATGTTAAAGCAGCTCAAAAGTTGCAT<br>GAGATTGATCCTCTTCAGAAGCCAAAGCAAGATAGATACGCTCTTAGAACATCTCCTCAATGGCTAGGCCCACAGAT<br>TGAAGTGATTCGTGCTGCGACGAAAATGATCGAGAGAGAGATCAACTCTGTGAATGATAATCCATTGATTGATGTTT<br>CAAGAAGTAAGGCGCTACATGGAGGCAATTTCCAGGGGACCCCAATTGGTGTTTCCATGGATAACACACGTCTTGC<br>CATTGCCTCCATTGGTAAACTCATGTTTGCACAATTCTCCGAGCTGGTTAATGACTTCTACAACAATGGATTGCCTTC<br>AAATCTTTCTGGCGGTCGCAATCCAAGCTTGGATTATGGATTCAAGGGTGCCGAAATCGCGATGGCATCTTACTGTT<br>CTGAACTCCAATTCCTTGCCAATCCTGTCACCAATCATGTCCAGAGCGCTGAGCAACACAACCAAGATGTGAACTC<br>TTTAGGCCTCATTTCTTCAAGAAAAACAGCTGAAGCTGTTGATATATTGAAGCTCATGTCCTCTACTTATTTAATTGC<br>TCTATGCCAAGCCATTGACTTGAGGCATTTGGAGGAGAACTTAAAAAACACTGTGAAGAACACAGTAAGTCAGTT<br>GGCCAAGAAACTCCTGACAATGGGATTCAATGGCGAACTTCACCCATCAAGATTCTGCGAAAAAGACTTGATCAA<br>AGTTGTGGACAGGGAATACGTTTTTGCATACATTGACGATCCCTGCAGTGCAACCTACCCATTAATGCAGAAACTAA<br>GGCAAGTCCTAGTTGAACATGCCATGGCCAATGGTGAAAAGGAGAAAAACTCAAGCACTTCAATTTTCCAGAAGA<br>TTGCAGTCTTTGAGGAGGAACTGAAAACCCAGCTTCCCAAAGATGTCGAAAGCACAAGGATCGAAATTGAGAATG<br>GAAATGCAGCAGTTCCAAACAAGATCAAGGAATGCAGGTCCTATCCATTATACAAATTTGTGCGAGAAGAACTTGG<br>AACAAGTTTTCTAACTGGTGAGAAAGTAAGGTCACCGGGTGAGGAATTTGACAAAGTTTTCTCAGCAATGTGTGC<br>AGGAAAGTTGATTGATCCCATGATGGAATGTTTAAAGGAGTGGAATGGAGCTCCTCTTCCTCTCTCCTAG
MiPAL1 promoter	CATAAGAATCATGCAAGCCATACAGAGACACAAAACTTTTTATGTAAATTTCTGGTGTAATAAATTTCTTTTTCCCTG<br>TAACCATGCAACTGTGATGTGGTTGACTAATTATTTTTCTTATATAAGATTTCAAACTATATTAAATATGTAAAATTTTT<br>AATTGATTAATTAATTTTATAATTATTTTATAAAATAAATTAAAATTTTAATTTTAAAATTTTGTCAGAAAAATCTTTACT<br>TTCTTATGCTTAAAATTCACTTTATTATCACTCTAATGATCAAGAAAATTAAGATTTTAGGTGCTATTATTATTATTTTT<br>GGTCACTTTCCCAATCCAAATTGAAATGCTAAATCTTGAAGTGAACAACTTCACTACGTTGAAAATTTCAAAACAG<br>ACCAATAATACGTCTCTTTCACCCAGTTTAGGGCACCATATGCAACTAATCTAATCCACCCCATTTTTTTCCTTCAAAT<br>TATTTATTATAATATAAAAAGATCAATCTAGTAAGAATTAATCAATGATCATTTGACTCATTTCATACCCAACAAAATAT<br>AAAATCTAACCTAATTTTTCGTATTAAAGTGAAACAAAATATGTACCTCCCAAAATATATTAGGTTTTCAACTAGTGT<br>TTGAAGACATCAAATTTATGGTGGAACATCATCCATGGAAAAGAAAGAAAATCTTAAATTATTTTTCATAGAAAAAA<br>CCTTTGCTTAATATTTAGAAGATAAATATATTTTGGAATTTAATGTGGCATTTGACAGTTGGTTTGAATTAAATGCAAG<br>CTAAAAAATGGTTAAAATAAATTACTTTACCTAAAATTAGATTAGCAGCAGCCGCTAAGAAACCACATAACTCCTAA<br>TTAACCACAATCAATTAATTAGCTTAATTACCTGATAAGCTCCTTCTGCAGGGCGCCACCTTCCTTCGTTCGCCTATG<br>CGACGTCGCACCGAAGCCAGTGGTCACTCCGTAGCTATCCGTCCCTTTGTTCA
MiMYB8	ATGGCGTTTAATAGAAAAGAAGTGGATCGGATCAAGGGCCCGTGGAGCCCAGAGGAGGACGAGGCGTTACAACAG<br>CTTGTGCAAAAGCATGGCCCAAGAAACTGGTCGTTGATAAGCAAGTCAATTCCGGGGCGATCCGGCAAGTCTTGCC<br>GTCTCCGGTGGTGCAATCAGCTCTCTCCGCAAGTTGAGCACCGCTCCTTCACACCTGAAGAAGACGAGACTATATT<br>ACGCGCTCATGCTCGCTTCGGCAACAAGTGGGCGACAATCGCCCGGCTTCTCAACGGCCGTACAGACAACGCGAT<br>AAAAAACCACTGGAACTCGACCTTGAAACGGAAGTGTTCAGGTTCGATGGCAGAGGATGGTAATTTTATCAACTGT<br>AATGGGTACGACGGGGATTTAGGTCATAATAATCAAAATCAGCCGTTGAAAAGATCTGTAAGTGCAGGATCAGGTG<br>TGCCGCTGACCACGGGGCTTTACGTGAACCCGAGTAGTCCATCTGGATCTGACGTAAGCGATTCCAGCGTACACGT<br>ATTTTCGTCGAATCACAACATATTTAAACCCGTTGCGAGAGCCGGCGGTGTTAATGTGGATGTGAATGTAATCCCCG<br>AAACGACATCGTCGAGCAACGATCCGCCAACTTCACTGAGTCTATCACTTCCAGGCGCCGATTCAAGCGAAGATAC<br>GAACCAGAAGGCAGCGGAGTCAAGTAAGGCTATAAATACTAAGAGTTTGTTCCCTGAGAGTGGAGCGAAAGGCGG<br>TGGCTTTCTAGGGTTTAGTCAAGAGTTCATGGGAGTGATGCAGGAGATGATAAGGCAGGAGGTGAGGAATTACATG<br>GCTTCCCTTGAACAACAGAGAGGTGGAGTTTGTTATCAGAGTAGTGGCGGTGGCGGCGGCGACGGTGTCTTTAGA<br>AATGTCGCGCTGCAGAGGATCTGA
